# Nanoparticle-Integrated Hydrogels as Multifunctional Composite Materials for Biomedical Applications

**DOI:** 10.3390/gels1020162

**Published:** 2015-10-14

**Authors:** Marco Biondi, Assunta Borzacchiello, Laura Mayol, Luigi Ambrosio

**Affiliations:** 1 Dipartimento di Farmacia, Università di Napoli Federico II, Via D. Montesano 49, 80131 Napoli, Italy; E-Mails: mabiondi@unina.it (M.B.); laumayol@unina.it (L.M.); 2 Istituto per i Polimeri Compositi e Biomateriali (IPCB-CNR), P.le Tecchio 80, 80125 Napoli, Italy; E-Mail: direttore.dsctm@cnr.it; 3 Dipartimento Scienze Chimiche e Tecnologie dei Materiali (DSCTM-CNR), P.le Aldo Moro 7, 00185 Roma, Italy

**Keywords:** nanocomposites, nanoparticles, hydrogels, biomedical applications, nanocomposite hydrogels

## Abstract

This review focuses on the most recent developments in the field of nanocomposite hydrogels intended for biomedical applications. Nanocomposite hydrogels are hydrated polymeric networks with a physically or covalently crosslinked three-dimensional (3D) structure swollen with water, in the presence of nanoparticles or nanostructures. A wide array of nanomaterials (polymeric, carbon-based, metallic, ceramic) can be incorporated within the hydrogel network to obtain reinforced nanocomposite hydrogels. Nanocomposites represent a new class of materials with properties absent in the individual components. In particular, the incorporation of nanomaterials within a polymeric hydrogel network is an attractive approach to tailor the mechanical properties of the hydrogels and/or to provide the nanocomposite with responsiveness to external stimuli.

## 1. Introduction

**Figure 1:**
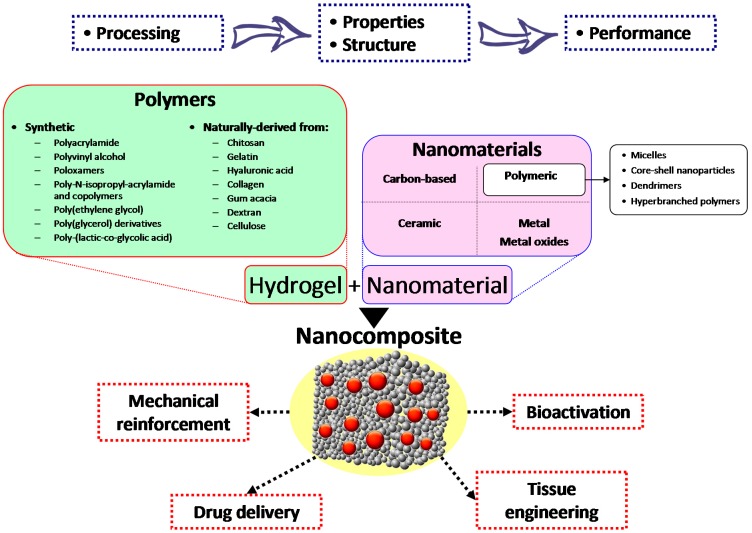
Processing parameters influence the structure and the properties of the NCH and, as a consequence, their performance in the medical field. Different polymers, both synthetic and naturally derived, can be used to produce the hydrogel, in which a variety of nanomaterials can be embedded. The resulting NCH can be used for a number of biomedical applications.

Hydrogels represent a class of soft materials, of synthetic and/or natural origin, of particular interest for biomedical applications such as tissue engineering, regenerative medicine, and controlled drug delivery [1,2,3,4,5,6,7,8,9,10,11] thanks to their physical, chemical, and biological properties compatible with those of biological tissues [12,13,14,15,16]. Hydrogels are physically or chemically cross-linked natural or synthetic three-dimensional (3D) networks, which can be cast into various shapes and retain high amounts of water (up to 4000% of their dry weight), although they are hardly hydrosoluble. Their water retention properties mainly arise from the presence of hydrophilic groups, such as amido, amino, carboxyl, and hydroxyl, in the polymer chains; the swelling degree depends on the polymer composition and, in addition, the cross-link density and nature. The water content of hydrogels creates a highly porous structure, a soft and elastic consistency, and a low interfacial tension in contact with water or biological fluids. These features make hydrogel’s properties closer to those of biological tissues than any other synthetic biomaterial. However, their application fields may be widened if a nanometric phase is embedded within the hydrogel’s matrix [2]. Thus, recent advances in hydrogel technology have led to the development of nanocomposite hydrogels (NCHs), also named nanocomposites, for biomedical applications; currently there is significant and increasing research interest in their development (Table 1). NCHs are hydrated polymeric networks with a physically or covalently crosslinked 3D structure, swollen with water in the presence of nanoparticles or nanostructures, that can be covalently or non-covalently immobilized in the matrix (Figure 1).

**Table 1:** Number of publications per year dealing with nanocomposite hydrogels (NCHs). As can be seen, since 2005 the scientific community devoted increasing interest to NCHs in the biomedical field. PubMed was used as the biomedical bibliographic database.

Publication Date	2002	2003	2004	2005	2006	2007	2008	2009	2010	2011	2012	2013	2014	June 2015
Number of publications	1	1	2	1	7	11	18	21	27	29	47	75	58	36

Research trends are currently focused on the incorporation of many nanoparticulate systems such as carbon-based nanomaterials, polymeric nanoparticles, ceramic nanoparticles, and metal/metal-oxide nanoparticles into the hydrogel network so as to obtain NCHs [17]. The interaction of these nanosystems with the polymeric chains of the hydrogel structure results in the peculiar properties of the nanocomposite absent in the individual components [17,18,19]. Nanoparticle addition may reinforce the starting hydrogels and provide the NCHs with responsiveness to external stimuli such as mechanical, thermal, magnetic, and electric. In particular, the nature of the nanosystems incorporated in the starting hydrogel determines the kind of stimuli to which the NCH is responsive. Here we will discuss the NCH properties and applications, classifying them on the basis of the chemical nature of the embedded nanomaterials summarized in Figure 1.

## 2. Carbon-Based Nanocomposite Hydrogels

**Figure 2:**
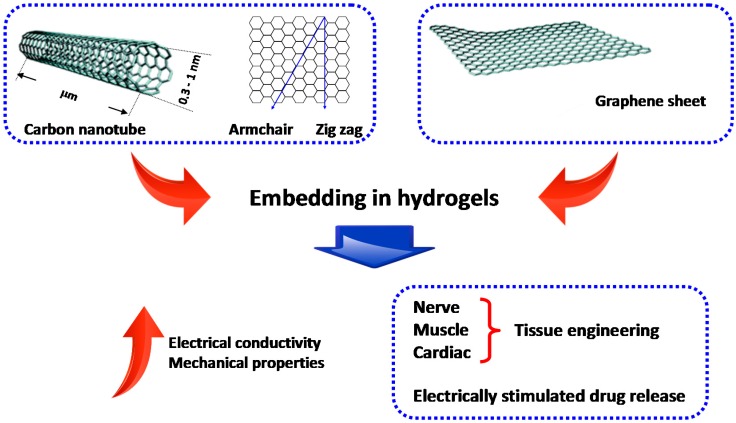
Nanocomposite hydrogels (NCHs) from carbon-based nanomaterials such as carbon nanotubes (CNTs) and graphene. CNTs exist in different atomic configurations (namely armchair and zig-zag) and architectures (single- and multi-walled) and can be chemically modified to enhance their hydrophilicity and, therefore, their interaction with the surrounding hydrogel. The addition/conjugation of CNTs and graphene derivatives provides NCHs with improved mechanical properties and electrical conductivity. For these reasons, NCHs embedding carbon-based nanomaterials can be potentially used for numerous applications, such as tissue engineering of electrically conductive tissues along with electrically-stimulated drug delivery.

Carbon-based nanomaterials such as carbon nanotubes (CNTs) and graphene are being used to provide conventional hydrogels with improved mechanical and electrical properties [20]. In particular, both CNTs- or graphene-based NCHs (Figure 2) are being studied for applications such as actuators, biosensors, tissue engineering scaffolds, drug delivery, and biomedical devices [18,21].

CNTs exist in the multi-wall or single-wall forms. Generally, CNTs are hydrophobic because of their strong π–π self-interactions, which in turn limits the interactions with the hydrogel network and favors the spontaneous formation of aggregates. These issues have been circumvented by surface modifications of CNTs by polar groups such as amines, hydroxyls, and carboxyls [22]. Due to their high electrical conductivity, NCHs reinforced with CNTs can be used to engineer electrically conductive tissues such as nerves, muscles, and cardiac tissues [23,24]. Graphene is a two-dimensional (2D) nanomaterial and is an excellent conductor of heat and electricity. Graphene solubility in physiological conditions and interaction with hydrophilic polymers can be ameliorated by its transformation into graphene oxide (GO) in an acidic milieu [25].

CNTs and GO, in addition to being physically dispersed in hydrogels, can be covalently conjugated to hydrogel polymer chains, thus promoting the transfer of the mechanical strength through the cross-linked network. For example, it has been estimated that the mechanical strength of a polymer matrix increases by an order of magnitude by introducing <1% of chemical bonds between CNTs and matrix [26]. Actually, the mechanical properties of a biomaterial are crucial and need to be properly tailored on the basis of the specific biological tissue to be replaced/repaired. Moreover, a scaffold for tissue engineering should be able to mimic the biological milieu of the extracellular matrix (ECM) so as to guide cell differentiation in a manner dependent on their stiffness. In this context, hybrid NCHs based on reinforced methacrylated gelatin (GelMA) have been recently produced as analogues of the ECM [27]. Multiwall COOH-functionalized CNTs were coated with GelMA by using the hydrophobic interactions between the polypeptide chains of GelMA and the sidewalls of the nanotubes. Moreover, it was also possible to photopattern the CNT-GelMA hybrid hydrogel and controllable dimensions and shapes could be attained. It was also shown that a CNT amount as low as 0.5% led to a threefold increase in the hydrogel tensile modulus [28]. Besides, CNT-GelMA hydrogels allowed a strong alignment of cardiomyocytes and the formation of tight intercellular junctions. It is interesting to note that, in the presence of CNTs, the external voltage needed to induce the cell beating was significantly reduced, thanks to the increase of electrical conductivity caused by CNTs. In more detail, a more stable spontaneous beating behavior was observed when cardiac tissues were cultured on CNT-GelMA and, furthermore, the beating rate was on average three folds higher than those measured from tissues cultured on CNT-free GelMA (~70 *vs.* 23 beats per minute on Day 6).

In general, by functionalizating CNTs with polymers, it is possible to promote the dispersion of carbon-based nanosystems and their physical interactions with the surrounding hydrogel [29].

Carbon-based NCHs have also been exploited for controlled drug delivery applications. In this context, CNTs and GO were used to reinforce hydrogels made up of natural/synthetic materials such as carboxymethyl guar gum, poly(acrylamide) (PAAm), and poly(vinyl alcohol) (PVA). Those hydrogels, despite their recognized biocompatibility, biodegradability, and biological recognition, lack the mechanical strength to control the release rate of loaded drug(s). When required, carbon-based nanomaterials are also added to confer an electrically triggered drug release. As an example, multiwalled CNTs have been proposed to reinforce hybrid hydrogels based on carboxymethyl guar gum for the transdermal delivery of diclofenac sodium [30,31]. Drug release was found to be slower with an increasing CNT content within the NCH. Nonetheless, the low permeability of the skin restricts the utility of this approach and major research is devoted to develop methodologies to increase the delivery of drugs across this barrier.

In the context of electrically triggered drug delivery, PVA-based NCHs containing reduced graphene oxide (rGO) for the release of lidocaine, a hydrophilic drug, have been explored. The delivery of the drug is based on the migration of the electrically charged drug toward the oppositely charged electrode. Higher rGO content was correlated to a more negative charge of the rGO-PVA polymeric network and to a faster release rate of lidocaine [32].

In another recent study, GO sheets were functionalized by peroxidation induced by radiation, to obtain graphene peroxide (GPO). The resulting GPO was conjugated to PAAm gel to produce NCHs. The chemical conjugation of GPO caused an increase in tensile strength and elongation increased by 900% and 500% after the addition of 3 mg/mL of GPO, compared to conventional hydrogels [33]. By this approach it is in principle possible to obtain tissues able to withstand prolonged mechanical stresses.

In summary, the addition/conjugation of CNTs and graphene derivatives to hydrogel matrices allows us to obtain NCHs with improved mechanical and electrical properties. However, the actual usefulness of carbon-based nanomaterials as tissue substitutes, scaffolds for tissue engineering, or matrices for controlled drug delivery is yet to be ascertained because of concerns about their *in vivo* biocompatibility, which has not yet been fully assessed. Indeed, various studies have been performed on carbon-based materials with many cell lines [34,35,36,37], and contradictory results have been obtained. For example, GO could support the proliferation and adhesion of kidney cells, osteoblasts,, and human embryonic stem cells [38,39]; on the contrary, other studies revealed a concentration-dependent cytotoxic activity of GO against fibroblasts [40], and a genotoxic effect against mesenchymal stem cells (hMSCs) [41]. These puzzling findings have been partially explained considering that the oxygen groups, preparation methods of GO, size, charge, and the structural defects of graphene affect the *in vivo* and *in vitro* interactions along with the overall biocompatibility [25].

## 3. Polymeric Nanoparticle-Based Nanocomposite Hydrogels

**Figure 3:**
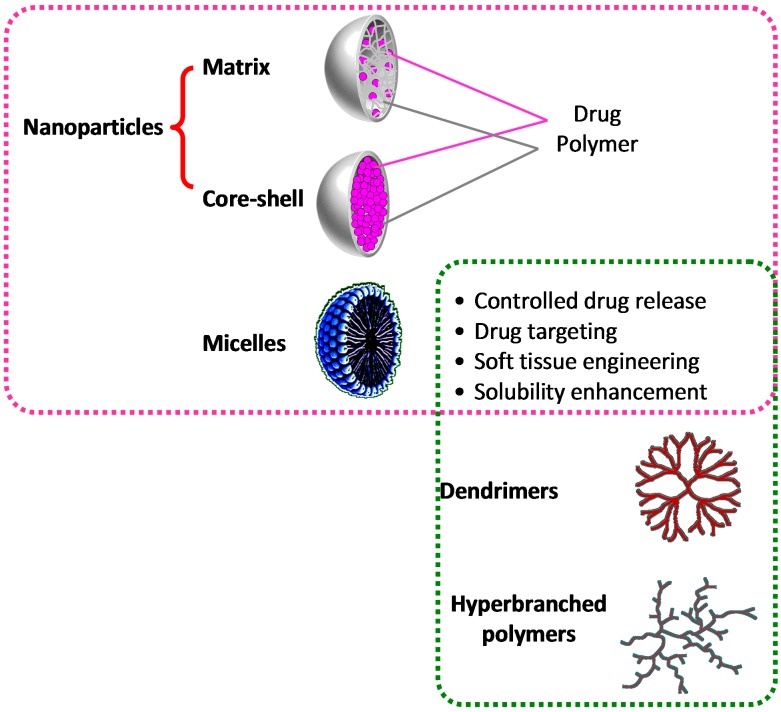
Nanocomposite hydrogels based on polymeric nanoparticles. Particle inclusion in hydrogels allows an increase of hydrogel mechanical properties, along with the possibility to control drug release rate. Amphiphilic (macro)molecules such as micelles, dendrimers, and hyperbranched polymers can also act as solubility enhancers of sparingly soluble drugs.

Among the manifold nanomaterials that can be incorporated into hydrogels, a variety of polymeric nanoparticles have also been used, mainly aiming to endow the final NCH with controlled drug release ability, along with the mechanical reinforcement [42]. Actually, the concept of polymeric nanoparticles [43] includes a number of other different systems such as micelles [44], core-shell particles [45], dendrimers [46], and hyper-branched polymers [47] (Figure 3).

The very nature of conventional hydrogels hampers the loading of hydrophobic drugs and, in this regard, NCHs, incorporating polymeric nanoparticles, can help to overcome this issue. For example, a micelle-based, NCH-encapsulating erythromycin, a hydrophobic antibiotic drug, has been recently developed by Liu *et al.* [48]. The drug has been encapsulated in Pluronic F-127 diacrylate macromer micelles and the hydrogel has been obtained by photopolymerization under a low-intensity UV light. Pluronics are triblock copolymers made up of poly(ethylene oxide)–poly(propylene oxide)–poly(ethylene oxide) (PEO–PPO–PEO), displaying amphiphilic properties due to the presence of hydrophilic EO and hydrophobic PO segments on polymer backbone and therefore widely used as solubility enhancers in the drug delivery field [49]. The produced NCH allowed for efficient loading and a sustained release of the drug. In a similar vein, many chemotherapeutic drugs are sparingly water soluble, and this is related to their unwanted noxious side effects. For instance, paclitaxel has been loaded within a thermosensitive micelle-hydrogel hybrid system based on Pluronic F-127 and carboxymethyl chitosan to reduce the dangerousness of the drug and to improve its water solubility. The system, intended for local chemotherapy, was cross-linked with glutaraldehyde and *in vivo* studies revealed a decrease in the tumor progression rate and a reduction in side effects compared with free paclitaxel [50].

Additionally, dendritic polymers can encapsulate drug molecules into their interior cavities or form polymer–drug complexes/conjugates through a host–guest chemistry and, furthermore, they can be used to reinforce the hydrogel network by covalent or non-covalent interactions [51]. Among dendrimers, poly(amidoamine) (PAMAM), made of repetitively branched subunits of amide and amine, exhibit high biocompatibility probably because they resemble the chemical structure of globular proteins. In a recent study PAMAM nanoparticles have been physically integrated into collagen scaffolds leading to a significant increase of scaffold mechanical properties and to a subsequently enhanced proliferation of human conjunctival fibroblasts in the hydrogels [52]. In another example, hyper-branched polyesters with a globe-like nanostructure have been employed to produce NCHs by UV photopolymerization. They have been shown to possess a controlled porosity and could encapsulate dexamethasone acetate, a lipophilic drug, with high efficiency due to their globe-like amphiphilic nanostructure. The resulting NCHs allowed a one-week release of the drug, which cannot be attained with a conventional control hydrogel [53]. These NCHs can be applied as systems that require porous structures with controlled drug delivery properties, such as scaffolds for tissue engineering [54]. In this context, a hydrogel scaffold made from a tri-block copolymer consisting of a poly(ethylene glycol) (PEG) core and methacrylated poly(glycerol succinic acid) dendrimer terminal block has been evaluated for soft tissue engineering applications. Depending on dendrimer concentration, hydrogel stiffness and hydration/degradation features could be tailored. In particular, the obtained NCH displayed a transition from primarily elastic behavior (loss angle δ~6°) at the lowest macromer concentration to a more viscoelastic behavior (loss angle δ~71°) at higher macromer concentrations and, correspondingly, the compressive modulus increased from ~4 KPa to ~34 KPa. Furthermore, the obtained NCH proved suitable for cartilage repair since encapsulated chondrocytes were able to synthesize neo-cartilaginous material containing proteoglycans and type II collagen [55]. In a recent work, hyperbranched polyglycerols (HBP) have been crosslinked by a one-step gelation via biomimicking mineralization, without organic solvents or catalysts [56]. The resulting NCH formed a 3D mesoporous network, and their mechanical properties could be easily tailored by regulating the amount of the precursor. HBP solutions possess a very low viscosity and, therefore, HBP-based NCHs can also be formulated at high concentrations (tens of percent). These nanosystems are optically transparent in both the wet and the dried state and hold great promise for applications as optical devices.

## 4. Ceramic Nanoparticle-Based Nanocomposite Hydrogels

**Figure 4:**
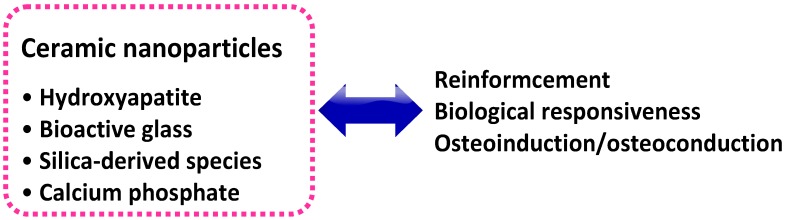
Nanocomposite hydrogels from ceramic nanoparticles. The inclusion of these nanomaterials allows a surprising reinforcement of the hydrogel. Furthermore, since most of these inorganic nanoparticles are made of minerals with a crucial role in the normal homeostasis of living tissues, they can provide the NCH with a biological responsiveness.

Several advanced NCHs can be obtained by combining inorganic ceramic nanoparticles with natural or synthetic polymeric hydrogels. A wide range of bioactive nanoparticles, such as hydroxyapatite (HAP), synthetic silicate nanoparticles, bioactive glasses, silica, calcium phosphate, glass ceramic, and b-wollastonite, can be used to this aim (Figure 4) [57]. Ceramic nanoparticles can reinforce the hydrogel, taking advantage of their high mechanical strength; furthermore, because they are made of minerals with a crucial role in the normal homeostasis and turnover of human tissues, they can also provide the final NCH with favorable biological cues [58]. Both these features are compelling reasons for their use in the field of tissue engineering and regenerative medicine. As a matter of fact, these properties of ceramic nanoparticle-based NCHs are cardinal to fulfilling the conflicting requirements of hard tissue engineering and regenerative medicine applications. Indeed, widely used scaffolding materials of natural origin such as polysaccharides (e.g., chitosan, CHI, or hyaluronic acid, HA), despite their attractive features such as biodegradability/biocompatibility, low toxicity, and low manufacture/disposal costs, generally lack mechanical and chemical stability and, therefore, their use as scaffolding materials as such is often compromised [59]. On the other hand, synthetic hydrogel materials such as PEG are bio-inert and, as such, are unable to impart an optimal milieu to endorse cell adhesion and tissue development; therefore, some bioactivation strategy must be taken in this sense [60]. In both cases, the addition of ceramic nanoparticles to obtain NCHs can be helpful to fulfill these needs.

HA is a naturally occurring linear polysaccharide and a primary constituent of ECM of human connective tissues [61,62]. HA is involved in diverse *in vivo* functions such as arthritis joint lubrication and control of soft tissues’ viscoelastic properties, and also in important cell functions such as cell motility and adhesion to the cell matrix [63,64]. To improve the mechanical performance of HA scaffolds, HA-based hydrogels have recently been reinforced with calcium and silica nanoparticles [65,66]. Bisphosphonate-functionalized HA hydrogels have been reinforced by reversible bonds with calcium phosphate nanoparticles [65]. The obtained NCHs displayed the capacity for self-healing as well as adhesiveness properties to mineral surfaces such as enamel and hydroxyapatite. Most importantly, these non-covalently cross-linked NCHs are surprisingly robust yet biodegradable upon extensive *in vitro* and *in vivo* testing and show bone interactive capacity evidenced by bone ingrowth into material remains. The second example of nanohybrid hydrogels consists of a cross-linked HA matrix including different amounts of silica-derived species. This inorganic filler phase controls the mechanical and swelling properties of cross-linked HA hydrogel, therefore making this NCH suitable for tissue engineering application, in which scaffold properties need to be modulated according to the specific tissue to be replaced.

Another natural polymer widely used in the biomedical field is CHI, because of its excellent biocompatibility, biodegradability, non-toxicity, and wound healing properties [67]. Aranaz and co-workers have recently reported a novel process method for the formation of NCH, namely ice segregation induced self-assembly (ISISA), based on urease-assisted hydrolysis of urea to produce CHI gels with a homogeneous and tailored 3D network structure. This process, applied to CHI solutions containing calcium phosphate salts, promoted the precipitation of nanoparticulate amorphous calcium phosphate and the gelation of CHI under mild conditions. The resulting NCH, with controlled properties, is suggested for its use as tissue engineering substrate [68]. In another report, calcium phosphate salts (CPS) and bone morphogenetic protein 2 (BMP2) have been immobilized, combined or alone, into CHI scaffolds by ISISA process. The obtained CHI-based NCH possessed the controlled release properties of BMP2, with a preserved osteoinductivity of the protein; furthermore, the NCH displayed interesting osteoconductivity features [69].

Among synthetic materials, PEG is also widely employed in tissue engineering thanks to its recognized biocompatibility. In a recent application, HAP was incorporated within a PEG matrix to confer elastomeric properties to the NCHs [70]. The addition of HAP to the polymeric network imparted elastomeric properties, enhanced mechanical strength, and improved the physiological stability of the NCH networks. For a starting 15% PEG hydrogel, the addition of HAP from 0% to 5% tripled the fracture stress and toughness of the obtained NCH, and its ultimate strain increased by 20%. Moreover, the addition of HAP resulted in enhanced osteoblast cell adhesion characteristics when compared with hydrogels made of PEG alone. Similar results were obtained when HAP was replaced with silica nanoparticles [71]. Due to their enhanced bioactivity and higher mechanical strength, these NCH networks (PEGHAP and PEG-Silica) can be used as injectable fillers for orthopedic applications [72].

## 5. Metal- and Metal Oxide-Based Nanocomposite Hydrogels

NCHs based on metal and metal oxide nanoparticles represent a promising new class of biomaterials since they possess several intriguing properties such as antimicrobial activity and responsiveness to electrical/magnetic stimuli and/or to light. Metallic nanoparticles mainly include noble metals such as platinum (Pt), gold (Au), and silver (Ag), or other metals such as cobalt (Co) and nickel (Ni), whereas metal oxide nanoparticles include iron oxide (Fe_3_O_4_, Fe_2_O_3_), titania (TiO_2_), alumina (Al_2_O_3_), and zirconia (ZrO_2_) [73].

Metallic nanoparticles can provide NCHs with antimicrobial activity since they are able to bind non-specifically to bacterial membranes, therefore inducing structural changes to bacteria, which allows for increased membrane permeability [74]. Moreover, metal/metal oxide nanoparticles stand out for their ferromagnetic and conducting/semi-conducting properties, thus providing the NCHs with electrical and magnetic properties that can be suitable for biomedical applications such as tissue regeneration.

In the context of antimicrobial materials, the intensified interest in NCHs with silver nanoparticles is due to their high antimicrobial effect, as recently reviewed by Dallas *et al.* [75]. Ag-based NCHs have been used as the functional coating in dental filling applications and also in wound and burn dressing to prevent infections. Different naturally occurring materials such as CHI, gum acacia, dextran, and gelatin, or synthetic materials such as PAAm, poly(acrylic acid) (PAA), *N*-isopropylacrylamide (NIPAAm), methyl methacrylate (MMA), and PVA have been used to incorporate metallic nanoparticles within the hydrogel matrix, thus obtaining NCHs with antimicrobial properties that are basically not harmful to healthy cells [76,77,78,79,80,81,82] Hydrogel-based substrates able to respond to magnetic and electrical fields are fundamental for the formation of tissues and in particular for those that require the propagation of electrical signals, such as nerves and muscles. For example, the incorporation of Au nanowires within alginate hydrogels has allowed us to improve the electrical conductivity between adjacent cardiac cells. Tissues grown on these composite matrices were thicker and better aligned than those grown on pristine alginate and, when electrically stimulated, the cells in these tissues synchronously contracted. The NCHs have shown an ability to be engineered as cardiac patches for treating damaged heart tissue after a heart attack [83]. Moreover, NCHs incorporating metal oxide nanoparticles can also enhance the bioactivity of hydrogels. In particular, nanoparticles of HAP and titania, entrapped within a polymeric matrix based on poly(l-lactic-*co*-glycolic acid) (PLGA), can enhance osteoblast adhesion and proliferation [84].

**Figure 5:**
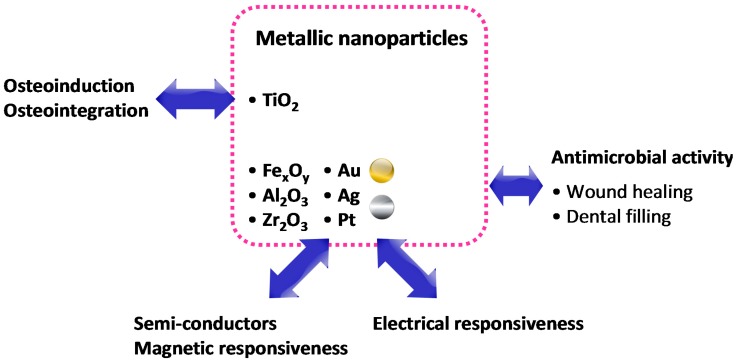
Nanocomposite hydrogels from metallic nanoparticles. The inclusion of metallic nanoparticles within hydrogels also allows us to obtain NCHs with electrical/magnetic responsiveness. In addition, noble metals such as silver also possess antimicrobial activity, and are therefore interesting for wound dressing.

Besides using the metallic or metal-oxide based nanocomposite hydrogels for the abovementioned applications, they are also explored for biosensing, diagnostic and bioactuation applications, and stimuli-responsive controlled drug release [73]. In the last field, Gaharwar *et al.* demonstrated that an NCH based on magnetic nanoparticles, covalently conjugated with thermoresponsive hydroxypropyl cellulose (HPC), can remotely interact with external magnetic fields in order to obtain stimuli-responsive hydrogels. In particular, when an external magnetic field was applied, the nanoparticles incorporated within the hydrogel network generate heat, thus resulting in coil-to-globule transition of the polymer chains and in the release of therapeutic agents from the nanocomposite hydrogel [85]. The response of nanoparticles to electromagnetic stimuli has also been applied to the preparation of light responsive hydrogels, which can be useful for drug delivery purposes and biotissue repair. A photoresponsive hybrid hydrogel loaded with core–shell, lanthanide-doped upconverting nanoparticles (UCNPs) has been used to convert near-infrared (NIR) light into UV light. When the hydrogel is irradiated with 980 nm light, photodegradation and subsequent release of the embedded therapeutics occur (Figure 5) [85]. The stimulus of light is also very interesting because it is instantaneous and can be delivered rapidly, easily, and with high efficiency. A light-responsive material can be used for cell instruction and drug delivery purposes. Porous CHI substrates containing gold nanorods, which can absorb incoming laser light, have been tested as laser-activatable adhesives. Under a near-infrared light, nanoparticles’ dispersion in CHI enables the activation of the polar groups of CHI strands, therefore enhancing tissue adhesion [87]. In a more recent example, similar NCHs based on CHI-containing gold nanorods were integrated with PCL–PEO–PCL micelles containing the drug to be released for on-demand release of a widely used antitumor drug, such as doxorubicin [88].

## 6. Future Perspectives

The incorporation of nanomaterials within polymeric hydrogels represents an attractive approach to tailor the mechanical properties of the hydrogels and/or to provide the NCH with responsiveness to mechanical, thermal, magnetic, and electric stimuli. Further studies have to be carried out to better understand the interactions, at different length scales, between the polymeric chains of the hydrogels and the nanophase and, in the case of drug delivery systems, between the interior part of the nanoparticles and the drug(s) loaded into them. In this context, the understanding of the relationship between structure and properties, at all scales from nano- to macro-, is crucial and will allow for custom design of NCHs’ physico-chemical and electrical properties, so as to tailor them for specific applications.

Furthermore, the integration of suitable biological cues within the NCHs may provide them with biological features, thus leading to an increasingly detailed design of the biomaterial to be used in the field of cell/drug delivery and tissue engineering. Additionally, the proper combination of multiple phases within a NCH network could allow us to better mimic the structure and properties of native tissues and design increasingly sophisticated, stimuli-responsive NCHs. All these strategies may direct the development of the next generation of NCHs.
